# Optimizing nitrogen application frequency to enhance the post‐flowering matter accumulation, yield and water/nitrogen use efficiency of densely planted and drip‐irrigated maize

**DOI:** 10.1002/jsfa.14411

**Published:** 2025-06-18

**Authors:** Dongping Shen, Linli Zhou, Keru Wang, Bo Ming, Ruizhi Xie, Jun Xue, Liang Fang, Tingting Zhang, Zhen Wang, Jinghui Yu, Xiaoxue Xu, Hui Jiang, Naijiao Ma, Guoqiang Zhang, Shaokun Li

**Affiliations:** ^1^ Key Laboratory of Oasis Eco‐Agriculture, Xinjiang Production and Construction Corps, College of Agronomy Shihezi University Shihezi China; ^2^ State Key Laboratory of Crop Gene Resources and Breeding, Key Laboratory of Crop Physiology and Ecology, Ministry of Agriculture and Rural Affairs, Institute of Crop Sciences Chinese Academy of Agricultural Sciences Beijing China; ^3^ Tongliao Agriculture and Animal Husbandry Development Center Tongliao China

**Keywords:** frequency of nitrogen application, integrated drip irrigation and fertilization, spring maize, water‐use efficiency, yield

## Abstract

**BACKGROUND:**

Maize yield stability is crucial for China's national food security. Conventional irrigation and nitrogen application methods have problems like low yield, inefficiency and environmental pollution. Optimizing water/fertilizer management is therefore imperative. This study reports on a field experiment conducted in Tongliao, Inner Mongolia (2020–2021) that used ‘Dika 159’ maize grown at a density of 9.0 × 10^4^ plants ha^−1^. Five nitrogen application frequencies were set up, 0 (F0), 2 (F2), 4 (F3), 6 (F4), 8 (F5) with drip irrigation, in addition to farmers’ one‐time basal and flood irrigation as the control (F1).

**RESULTS:**

Compared that of F1, the leaf area index of F4 increased by 5.05% at the VT (silk emergence) stage and by 73.01% at the R6 (maturity) stage, and the maximum leaf area duration appeared at the VT‐R3 (silk emergence–milk ripening) stage. The frequency of nitrogen application mainly affected the post‐anthesis photosynthetic rate of maize. F4 and F1 did not differ significantly in their pre‐anthesis matter accumulation, but F4's post‐anthesis matter accumulation was significantly higher, the maximum dry matter accumulation rate of F4 being 94.44% higher than that of F1. The six‐time nitrogen application resulted in the optimum yield (15.82–16.06 t ha^−1^) and physiological nitrogen‐use efficiency (PNUE; 7.87–7.44 kg kg^−1^), and its water‐use efficiency (WUE) reached 2.10–2.14 kg m^−3^, raising the yield by 65.75–69.84%, enhancing the WUE by 62.12–62.79% and improving the PNUE by 29.23–40.11%.

**CONCLUSION:**

A greater frequency of nitrogen application (six times) can prolong the functional period of maize leaves, slow leaf senescence, enhancing the post‐anthesis photosynthetic capacity of maize to bolster its post‐anthesis matter accumulation and kernel weight, improving the yield and water/fertilizer utilization rate. © 2025 The Author(s). *Journal of the Science of Food and Agriculture* published by John Wiley & Sons Ltd on behalf of Society of Chemical Industry.

## INTRODUCTION

The growing global population has worsened grain demand.^1^ To boost food production, more farmland, better agronomic management and precision technologies are thus needed.^2^ Dense planting is a therefore key solution.^3^ But cultivation at a higher density demands more water and fertilizer inputs. Water shortages pose a challenge to agricultural production, and how to reduce agriculture's water consumption and improve its water‐use efficiency are mounting concerns.^4^ Nitrogen fertilizer, widely used in modern agriculture, can enhance both crop yield and quality, yet its excessive use may have negative impacts. Improper nitrogen management leads to increased N_2_O emissions, can lead to substantial soil and water pollution and contribute to global warming,^5^ harming the environment. Maize is now China's largest food crop, and its high and stable yield underpins its national food security.^6^ However, conventional fertilization and irrigation methods have recurring issues, such as wasted water and fertilizer resources, low crop yields and low general efficiency. Accordingly, finding a way to increase maize yield while also improving water‐ and fertilizer‐use efficiency are urgent issues in agriculture.

In recent decades, farmers have often applied large amounts of nitrogen fertilizer to obtain high yields of maize. In northeast China's supplementary irrigation areas, farmers mostly use the ‘one‐time basal application and flood irrigation’ mode: fertilizing once during sowing or the vegetative period, which causes early‐stage fertilizer redundancy, nitrogen loss and volatilization and leaching, thereby polluting the soil and environment.^7^, ^8^ Drip irrigation with a split application of nitrogen fertilizer is an excellent way to improve water‐/nitrogen‐use efficiency, by not only reducing nutrient losses from runoff and volatilization^9^ but also minimizing water waste from improper irrigation.^10^ Delaying the nitrogen application closer to the crop's peak nitrogen‐demand period could also enhance nitrogen‐use efficiency.^11^ Previous studies have found that maize has weak nitrogen sensitivity before anthesis, so in general the soil‐available nitrogen suffices for its early growth. But post‐anthesis, nitrogen deficiency is the main factor affecting the yield of maize.^12^ Hence, applying nitrogen fertilizer during the late growth stage of maize could effectively improve its yield and nitrogen‐use efficiency.

Water and nitrogen are pivotal factors regulating the irrigated agricultural ecosystem.^13^ In different water/nitrogen management practices, changes in water–nitrogen dynamics can affect the partial factor productivity of nitrogen^14^ and water‐use efficiency.^15^ Standard irrigation and fertilization methods often increase the risk of nitrogen loss and result in more water wasted.^16^ Drip irrigation and fertilization integration technology is a water/fertilizer integrated mode of agricultural management.^17^ Compared with other irrigation technologies, drip irrigation and nitrogen application can reduce water/nitrogen losses and boost crop yields.^17^ Previous studies have shown that compared with traditional flood irrigation, drip irrigation conserves irrigation water, improves water‐use efficiency by 26.4% and increases the maize yield by 14%.^18^ Under the same nitrogen amount with drip irrigation, the split‐application of nitrogen can effectively reduce NO_3_ leaching while increasing maize yield.^19^ This suggests that drip irrigation combined with different nitrogen‐application strategies can substantially affect soil nitrogen loss,^20^ and influence both crop yield and water‐/fertilizer‐use efficiency.

Located in China's golden maize belt, Tongliao (Inner Mongolia) is home to an annual planting area of at least 130 × 10^4^ ha, responsible for about 5% of the national total maize output. Over 90% of the land is irrigated. Maize yield has risen with the implementation of integrated water–fertilizer irrigation technology,^21^ yet the fertilization technology has flaws. Most farmers use extensive water/fertilizer management practices: 60% rely on pre‐silking fertilization, usually with two or four drip‐irrigation fertilizations. This causes high nitrogen consumption early on and nitrogen deficiency later. Therefore, given the unreasonable application of nitrogen fertilizer in this area, whether using the integrated drip irrigation and water–fertilizer mode and setting a staggered fertilization schedule – applying the same amount but spread over longer times – can not only effectively improve the utilization rate of water and fertilizer, but also maintain yield, is an unresolved problem.

To address these concerns, the objectives of the study reported here were (1) to clarify the mechanism for improving maize yield and water/fertilizer utilization under differing nitrogen application frequencies; and (2) to determine the optimal number of times that nitrogen should be applied for high‐yield, densely‐planted, drip‐irrigated maize, thus providing guidance and support to agricultural water management. The findings will aid in spurring an agricultural transformation and promote food production sustainability, contributing to global food security and sounder agricultural development.

## MATERIALS AND METHODS

### Site description

**Figure 1:**
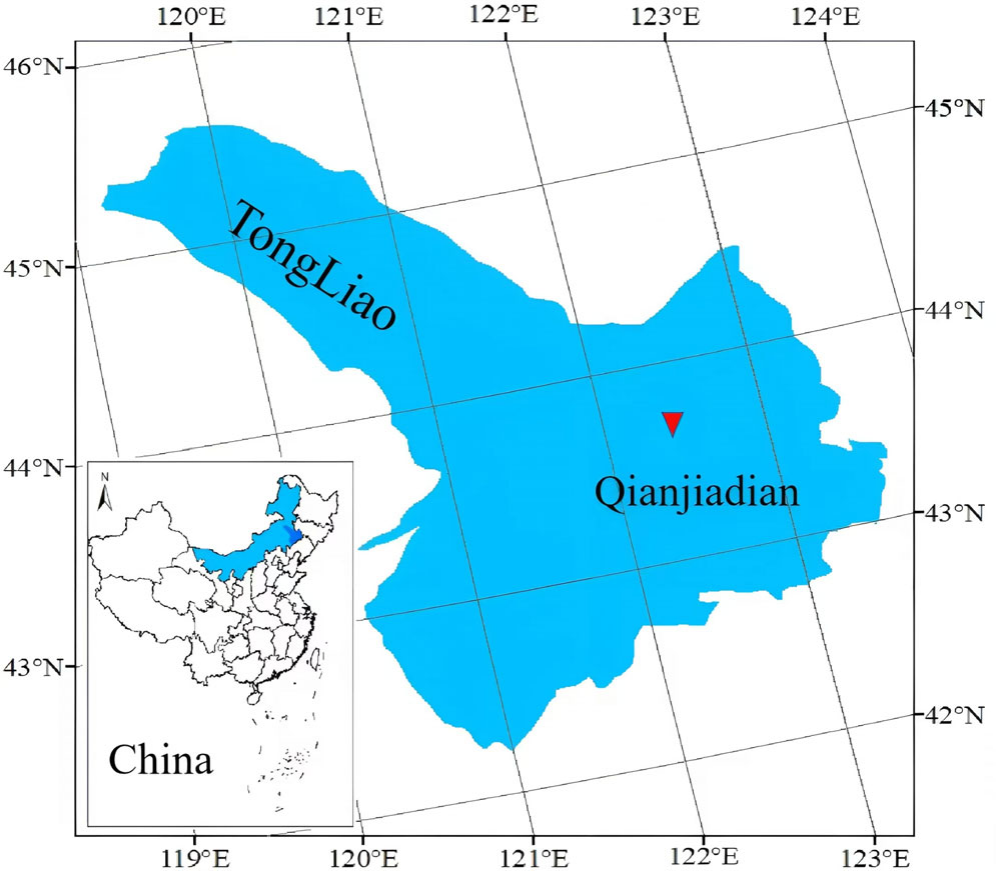
Experimental site location.

Field experiments were carried out in Qianjiadian Town, Tongliao City, Inner Mongolia (latitude: 43°43′ N, longitude: 122°30′ E, altitude: 174 m) in 2020 and 2021 (as shown in Fig. 1). The cumulative temperature of ≥10 °C ranged from 3000 to 3300 °C. The annual sunshine duration was between 2500 and 2800 h, and the frost‐free period was from 150 to 169 days. The rainfall and average temperature during the two‐year growth period of maize are presented in Table 1. The soil in the Qianjiadian experimental site was sandy clay loam, with 48.0% sand, 7.99% clay and 44.01% loam. The content of soil organic matter in the 0–60 cm soil layer was 23.88 g kg^−1^, the alkali‐hydrolyzable nitrogen was 87.5 mg kg^−1^, the available phosphorus was 6.92 mg kg^−1^, the available potassium was 196.9 mg kg^−1^ and the pH was 7.4.

**Table 1:** Precipitation and average temperature during the growing seasons of maize in 2020 and 2021

Month	Precipitation (mm)		Average temperature (°C)	
	2020	2021	2020	2021
May	7.2	4.8	17.3	19.5
June	123	28.8	23.7	21.8
July	65.9	105.4	26.1	25.6
August	61.0	154.6	22.6	21.4
September	101.9	32.8	16.3	16.9
Total or average	359.1	326.4	21.2	21.0

### Experimental design

The experiment had a single‐factor randomized block design. Five drip irrigation and fertilization frequencies were set, namely 0 times (no nitrogen fertilizer application, F0), 2 times (F2), 4 times (F3), 6 times (F4) and 8 times (F5). The one‐time basal application with flood irrigation by farmers (F1, CK) served as the control. The maize variety tested was ‘Dika 159’ (DK159), with a planting density of 9.0 × 10^4^ plants ha^−1^. During the entire two‐year growth period, 270 kg ha^−1^ of pure nitrogen, 120 kg ha^−1^ of pure phosphorus (P₂O₅) and 150 kg ha^−1^ of pure potassium (K₂O) were applied.^22^ Phosphorus and potassium fertilizers were applied once, as seed fertilizers. For the F2–F5 treatments, 50 kg ha^−1^ of pure nitrogen was applied as seed fertilizer; the remaining amount of nitrogen fertilizer was applied in equal amounts in installments during the growth period. The F0 treatment did not receive any nitrogen fertilizer. The nitrogen fertilizer in the F1 treatment was applied once, as seed fertilizer. The specific fertilization times are presented in Table 2.

**Table 2:** Nitrogen top‐dressing periods, nitrogen application rates and proportion of nitrogen applied before and after silking in the different treatments

Treatments	Nitrogen top‐dressing periods	Single application of nitrogen (kg ha^−1^)	Proportion of nitrogen application (%)		
			Base fertilizer	Before silking	After silking
F2(2次)	V8, VT − 5d	100	18.5	81.5	0
F3(4次)	V8, V12, VT − 5d, VT + 10d	50		61.1	20.4
F4(6次)	V8, V12, VT − 5d, VT + 10d, VT + 30d, VT + 40d	33.3		40.75	40.75
F5(8次)	V8, V12, VT − 5d, VT + 10d, VT + 20d, VT + 30d, VT + 40d, VT + 50d	25		30.6	50.9

### Experimental management

Seeding was carried out on 9 May and harvesting was done on 3 October in both years. The wide–narrow row planting method (40 + 80 cm) was adopted, and a shallow‐buried drip irrigation and fertilization integration system was used for irrigation and fertilization. The plot area was 72 m^2^ (10 m × 7.2 m), with three replications. The irrigation water for maize was groundwater, which was irrigated through the drip irrigation system. The irrigation quota for F1 was set to 450 mm, with flood irrigation carried out five times. The irrigation quotas for other treatments were set to 270 mm, with drip irrigation carried out eight times. Drip irrigation for seedling emergence technology was adopted. One day after sowing, except for F1, 45 mm of emergence water was drip‐irrigated to promote seed germination, ensure uniform and neat seedlings and improve the uniformity of the maize population. For F1, 90 mm of water was flood‐irrigated. Irrigation and fertilization started 54 days after sowing. All weeds, pests and diseases in the experimental field were effectively prevented and controlled.

### Measurement indices and methods

#### Leaf area index (LAI), leaf area duration (LAD) and photosynthetic rate (Pn)

Three maize plants were randomly sampled at the 6‐expanded‐leaf stage (V6), 12‐unfolded‐leaves stage (V12), silk emergence (VT), milk ripening (R3), wax ripening (R4) and maturity (R6) to measure the leaf area (LA). LA, LAI and LAD were calculated according to Eqns ((1), (2), (3), (4))–((1), (2), (3), (4)):

(1) $$\mathrm{LA}=0.5\;\times \;\mathrm{LL}\;\times \;\mathrm{LW}\;\left(\text{unexpanded leaf}\right)$$

(2) $$\mathrm{LA}=0.75\;\times \;\mathrm{LL}\;\times \;\mathrm{LW}\;\left(\text{expanded leaf}\right)$$

(3) LAI=Leaf area/Ground area

(4) $$\mathrm{LAD}=\left(\left(\mathrm{LAI}t_{1}+\mathrm{LAI}t_{2}\right)/2\right)\;\times \;\left(t_{1}-t_{2}\right)$$

In the formulas, LL is the leaf length (cm) measured from the leaf collar to the leaf tip, LW is the leaf width (cm) measured at the widest part of the leaf and *t* is the growth period time. On sunny days between 11:00 AM and 1:00 PM, the Pn of three representative ear leaves from each plot was measured using a CIRAS‐3 portable photosynthesis system at the VT, R3 and R4 stages.

#### Dry matter

Three maize plants were selected at V6, V12, VT, R3, R4 and R6 stages. The leaf, stem, sheath and female and male panicles were dried at 105 °C for 30 min, dried at 80 °C until constant weight was reached and were then weighed. The dried plant samples were weighed, ground into powder, passed through a 1 mm sieve and digested with H_2_SO_4_–H_2_O_2_. Plant nitrogen concentration was determined using a K9840 semi‐automatic Kjeldahl nitrogen meter. The characteristic values of the material accumulation rate, *t*_1_, *t*_2_, vm, tm and td, were calculated using Eqns ((5), (6), (7), (8), (9))–((5), (6), (7), (8), (9)):

(5) $$t_{1}=-1/b\;\times \;\ln\left(2+\sqrt{3}\right)/a$$

(6) $$t_{2}=-1/b\;\times \;\ln\left(2-\sqrt{3}\right)/a$$

(7) $$\mathrm{td}=t_{2}-t_{1}$$

(8) $$\mathrm{tm}=\ln\left(a/b\right)$$

(9) vm=b×k/4

In the formulas, *t*_1_ represents the starting time of the maximum accumulation rate, *t*_2_ represents the ending time of the maximum accumulation rate, vm is the maximum accumulation rate and tm is the duration of the maximum accumulation rate.

#### Grain yield

At physiological maturity, an area of 36 m^2^ (10 m × 3.6 m) from each plot was harvested manually, and the grain mass was measured. The plants and ears were counted, and the number of ears per plant was determined. Grain moisture content was determined with a portable moisture meter (PM8188A). Grain yield was determined at a 14% moisture content.

#### Nitrogen‐use efficiency

(10) Partial factor productivity of nitrogen:PFPN=GY/F

where PFPN is partial factor productivity of nitrogen (kg kg^−1^), GY is grain yield (t ha^−1^) and *F* is nitrogen input (kg ha^−1^).

(11) $$\text{Agronomic efficiency of nitrogen}:\mathrm{AEN}=\left(\mathrm{GYn}-\text{GY0}\right)/F$$

where AEN is agronomic efficiency of nitrogen (kg kg^−1^), GYn is grain yield (t ha^−1^) and Y0 is yield without nitrogen fertilization (kg ha^−1^).

(12) $$\text{Physiological efficiency of nitrogen}:\mathrm{PEN}=\left(\mathrm{GYn}-\text{GY0}/\right)/\left(\mathrm{Na}-\text{Na0}\right)$$

where PEN is physiological efficiency of nitrogen (kg kg^−1^), Na is nitrogen accumulation (kg kg^−1^) and Na0 is nitrogen accumulation without nitrogen fertilization (kg kg^−1^).

(13) $$\text{Recovery efficiency of nitrogen}:\mathrm{REN}=\left(\mathrm{Na}-\text{Na0}\right)/F$$

where REN is recovery efficiency of nitrogen (kg kg^−1^).

#### Water‐use efficiency

Maize actual evapotranspiration ETc (mm) was calculated during the growing season using the soil water balance equation as follows:

(14) ETc=I+Pe−Cr−Rf−Dp±ΔS

where *I* is the amount of applied irrigation water (mm); Pe is precipitation (mm); Cr is capillary rise (mm); Dp is percolation (mm); Rf is runoff (mm); and Δ*S* is the change in soil–water storage (mm).

In Eqn (14), Cr is considered to be zero, runoff is assumed to be insignificant as the field was flat and Dp is also considered to be negligible because the soil water content below 100 cm did not reach field capacity on any sampling date.

The water‐use efficiency WUE (kg m^−3^) was represented as the grain yield (GY, t ha^−1^) per unit of ETc (mm):

(15) WUE=GY/ETc×100

### Meteorological data

The meteorological data were automatically obtained from a Watchdog 2900 ET meteorological station (SPECRUM, USA) set up at the experimental site.

### Statistical analysis

Excel 2013 was used to organize the data and create tables, Origin 2022 was used for plotting and LSD (*P* < 0.05) test was used for multiple comparisons and significance analysis. ArcGIS were used for mapping plots.

## RESULTS

### Yield, yield composition, nitrogen‐use efficiency, evapotranspiration and water‐use efficiency

The year had no significant effect on the number of grains per ear, but it significantly influenced the number of harvested ears, grain weight, yield, PFPN, AEN, PEN, REN, ETc and WUE. Although the number of nitrogen applications had no significant effect on the number of harvested ears, it did significantly affect other crop parameters. The interaction between year and frequency of nitrogen application affected all those parameters (factors) except the number of harvested ears (Table 3). From 2020 to 2021, the F4 treatment exhibited no significant difference in the number of grains per ear when compared with F5, F3 or F2, but it was 17.41–17.50% higher than the control (F1). The yield and 1000‐grain weight of F4 and F5 were similar, yet significantly greater than that of F3, F2 and F1. In general, PFPN, AEN, PEN and REN all increased with more nitrogen applications. While the F4 treatment showed no significant differences from F5, its PFPN, AEN, REN and PEN were higher than those of F3, F2 and F1. Likewise, WUE increased with a greater frequency of fertilization, with F4 being similar to that of F5, yet higher than those of F3, F2 and F1. Increasing the number of fertilizations had a small impact on the number of grains per ear, whereas increasing post‐silking nitrogen application was able to increase 1000‐g weight, grain yield and water‐/fertilizer‐use rates.

**Table 3:** Maize yield, yield composition, nitrogen fertilizer utilization efficiency, evapotranspiration (ETc) and water‐use efficiency (WUE) under different nitrogen fertilizer treatments in 2020 and 2021

Year	Treatment	Harvest ear (10^4^ plant ha^−1^)	Kernels per spike	1000‐kernel weight (g)	Yield (t ha^−1^)	PFPN (kg kg^−1^)	AEN (kg kg^−1^)	PEN (kg kg^−1^)	REN (kg kg^−^)	ETc (mm)	WUE (kg m^−3^)
2020	F0	8.46a	313.45c	252.45e	6.01e	—	—	—	—	697.03e	0.86e
	F1	8.47a	402.75b	294.35d	9.81d	36.33d	14.07d	6.09d	0.23d	738.02d	1.32d
	F2	8.47a	468.21a	316.41c	11.99c	44.07c	21.81c	6.75c	0.33c	744.57c	1.59c
	F3	8.46a	474.14a	360.35b	13.27b	49.15b	26.89b	7.57b	0.36b	749.50b	1.77b
	F4	8.51a	472.85a	434.27a	16.26a	60.33a	37.22a	7.87a	0.48a	760.95a	2.14a
	F5	8.49a	473.61a	438.27a	16.09a	59.48a	36.26a	7.69a	0.49a	763.94a	2.11a
2021	F0	8.54a	305.54c	256.78e	5.89e	—	—	—	—	680.27e	0.87e
	F1	8.54a	395.43b	288.42d	9.35d	34.63d	12.81d	5.31d	0.24d	725.07d	1.29d
	F2	8.53a	463.57a	311.61c	10.54c	39.04c	17.22c	5.77c	0.29c	731.33c	1.44c
	F3	8.53a	464.07a	346.13b	12.09b	44.78b	22.96b	6.24b	0.377b	745.90b	1.62b
	F4	8.54a	466.64a	421.94a	15.88a	58.81a	37.00a	7.44a	0.50a	755.40a	2.10a
	F5	8.55a	470.12a	420.46a	15.82a	58.59a	36.78a	7.20a	0.51a	759.37a	2.08a
Source of variation											
Year		**	ns	**	**	**	**	**	**	**	**
Treatment		ns	**	**	**	**	**	**	**	**	**
Year × treatment		ns	**	**	**	**	**	**	**	**	**

Different letters indicate a statistical difference between means at *P* < 0.05. ns, not significant. **Significant at *P* < 0.01. Likewise for the following tables.

### LAI and LAD

**Figure 2:**
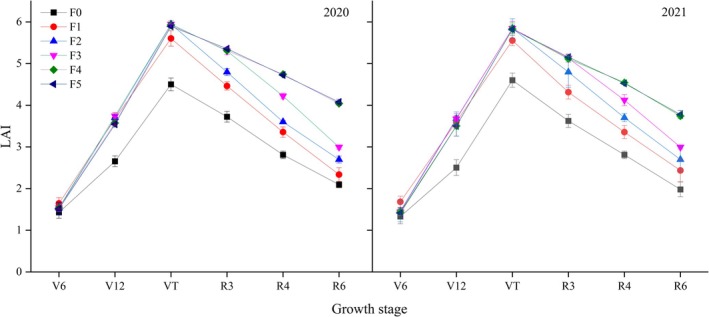
Changes in LAI of different treatments at different growth stages of maize, in both years (2020 and 2021). Along the *x*‐axis, V6 denotes the 6‐unfolded‐leaves stage, V12 denotes the 12‐unfolded‐leaves stage, VT denotes silk emergence, R3 denotes milk ripening, R4 denotes wax ripening and R6 denotes maturity. Different letters indicate statistically significant differences at the *P* < 0.05 level.

**Figure 3:**
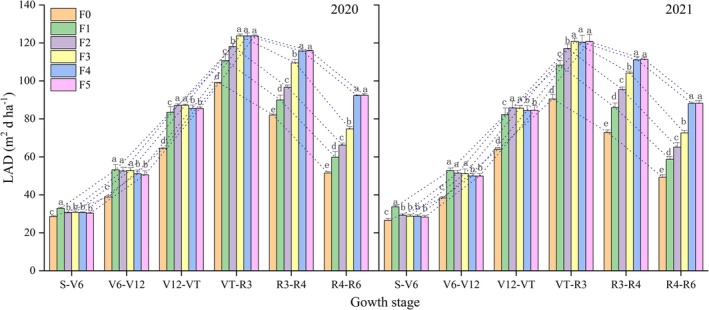
Changes in LAD of maize during its growth stages and under different treatments, in both years (2020 and 2021); S denotes the sowing period. The dashed line is the rate of increase and decrease in LAD at each stage.

The LAI and LAD of maize under the differing fertilization treatments in the 2 years varied across growth stages (Figs 2 and 3). Both plant traits showed a trend of first increasing then decreasing with progression through growth stages, generally peaking at VT (VT to R3) stage. In 2020 and 2021, during the V6 stage the LAI was significantly greater in F1 than in the other treatments; however, during the V12 stage, the LAI of F1 was indistinguishable from that of F2, F3, F4 and F5 treatments, yet it significantly exceeded that of the F0 treatment. At the VT stage, though the LAI of the F4 treatment was similar to that of the F2, F3, and F5 treatments, it was 5.42–5.89% significantly higher than that of the F1. During the R3 stage, the F4 treatment's LAI did not differ significantly from that of either the F3 or F5 treatment, but it was 6.46–10.63% and 18.56–19.05% significantly higher than in F2 and F1, respectively. During the R4 stage, while the LAI was similar between the F4 and F5 treatments, these values were 10.19–12.32%, 22.70–31.67% and 35.12–41.07% significantly higher than those of F3, F2 and F1, respectively. During the R6 stage, the LAI of the F4 treatment was on a par with that of the F5 treatment, albeit still significantly surpassing that of the F3 and F2 treatments, as well as F1.

At the S to V6 stages, F1 had an LAI significantly larger than that of other treatments. During the V6 to V12 stages, there was no significant difference in LAD between F1 and either the F2 or F3 treatment; but the LAD was significantly higher for F1 than for both F4 and F5. Afterward, the LAD of F1 began to decrease during the V12 to VT stages. The LAD of the F2 and F3 treatments was significantly higher than those of the F4 and F5 treatments and significantly higher than that of the F1 treatment. During the VT to R3 stages, there was no significant difference in LAD between F3 and the F4 or F5 treatment, but the F3 treatment's LAD was significantly greater compared with the F2 and F1 treatments. The LAD of the F2 treatment began to decrease during the VT to R3 stages. From the R3 to R6 stages, the LAD was similar between the F4 and F5 treatments, but exceeded that of he F3, F2 and F1. In the F3 treatment, LAD declined at the R3 to R4 stages.

The leaf growth rate (GR) during the S to VT stages and the leaf senescence rate (AR) during the VT to R6 stages were calculated (Table 4). Evidently, the F4 and F5 treatments had a higher GR, but a lower AR, when compared with the other treatments. Relative to F1, the GR of the F4 treatment was 19.23–22.67% higher and its AR was 34.51–38.79% lower. Increasing the frequency of nitrogen application can prolong the leaf functional period after silking, which effectively delays leaf senescence, enlarges the LAI and enhances the photosynthetic capacity of maize.

**Table 4:** Changes in the growth rate and aging of maize leaves under a differing frequency of nitrogen application

Year	Treatment	Growth rate (%)	Aging rate (%)
2020	F0	0.71	1.09
	F1	0.78	1.16
	F2	0.88	1.19
	F3	0.93	1.12
	F4	0.93	0.71
	F5	0.93	0.71
2021	F0	0.64	0.94
	F1	0.75	1.13
	F2	0.88	1.18
	F3	0.92	1.09
	F4	0.92	0.74
	F5	0.92	0.74

### Photosynthetic rate

**Figure 4:**
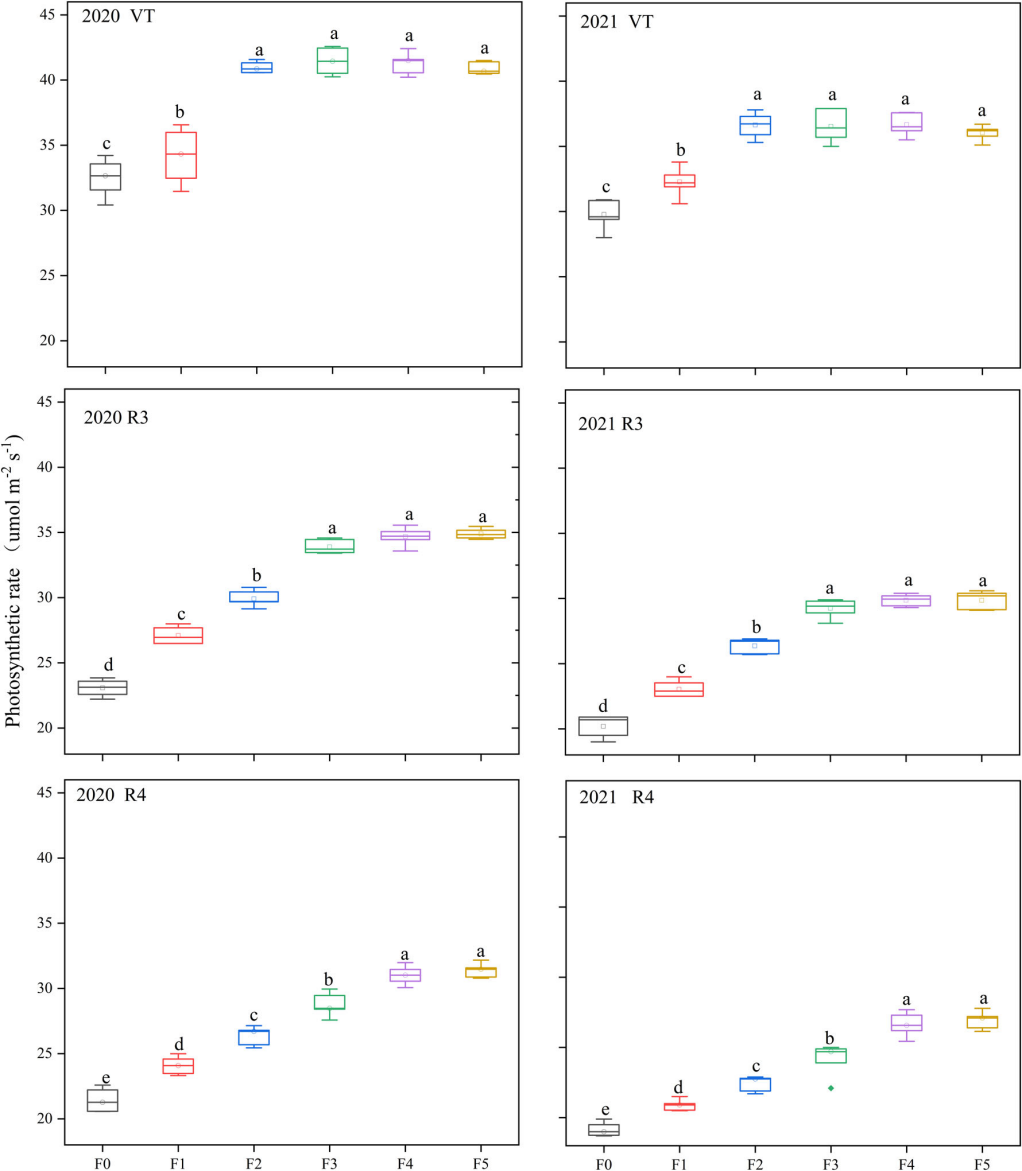
Boxplots showing the impact of different fertilization treatments on the photosynthetic rate of maize, in both years (2020 and 2021).

The frequency of nitrogen application affected the Pn of maize after silking (Fig. 4). At the VT stage, while the Pn of the F4 treatment was on a par with that of the F5 or F3 treatment, it was 12.99–20.8% significantly higher than that of the F1 treatment. At the R3 stage, the Pn of F4 remained similar to that of F5, but it was significantly higher than those of the F2 and F1 treatments, by 13.38–15.99% and 28.02–30.43%, respectively. At the R4 stage, the F4 treatment's Pn was indistinguishable from that of F5, yet 8.01–10.03%, 17.46–18.59% and 17.74–28.76% significantly higher than the 2‐year average of the F3, F2 and F1 treatments, respectively. To sum up, these results showed that increasing the frequency of nitrogen application enhanced the Pn after flowering, which enabled the accumulation of substances after flowering that further increased the yield of maize.

### Dry matter accumulation and distribution

**Figure 5:**
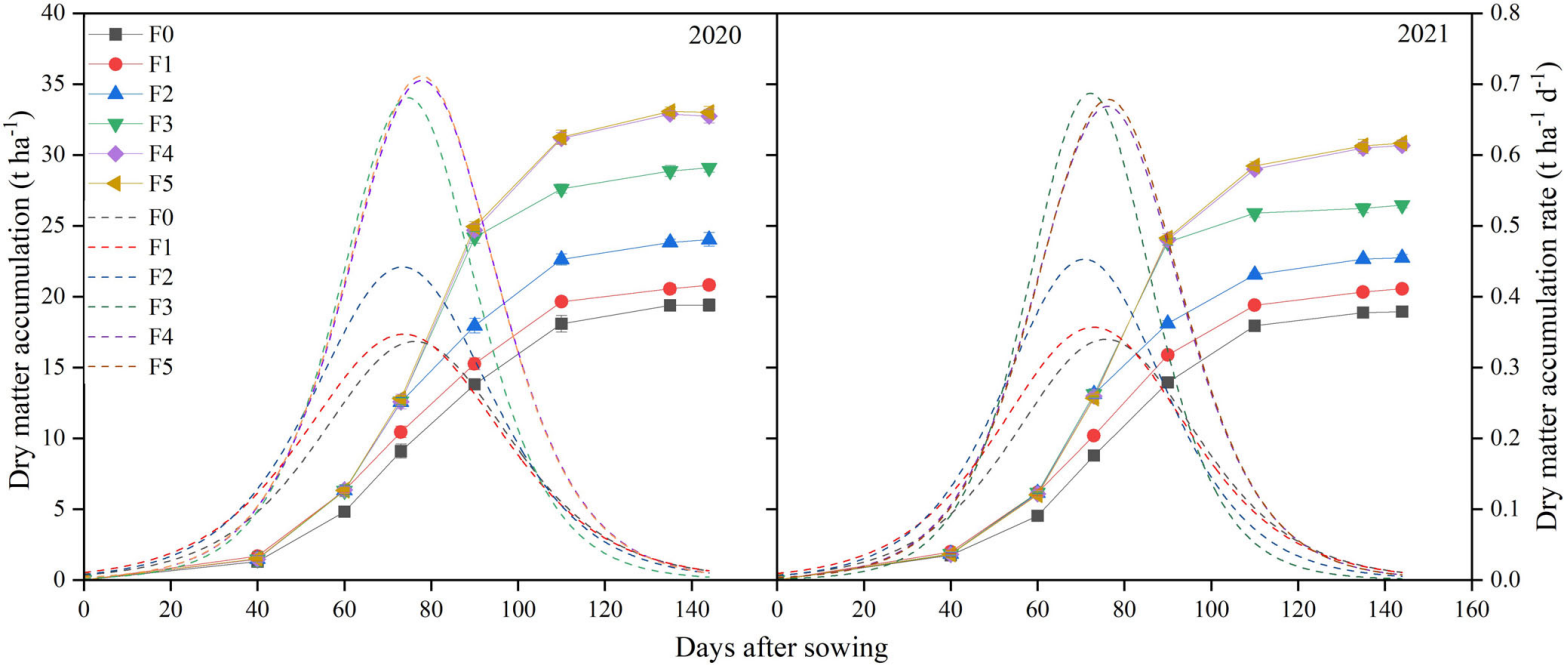
Effects of different irrigation and fertilization treatments on dry matter accumulation and dry matter accumulation rate in 2021 and 2022. The solid and dashed lines represent dry matter accumulation and dry matter accumulation rate, respectively.

The accumulated dry matter under the different fertilization treatments in the 2 years rose with more days since sowing. Although slow in the early and late phases, dry matter accumulation was rapid midway (Fig. 5). There was no significant difference in dry matter accumulation between the F4 and F5 treatments during each growth period. At 73 days after sowing, no significant differences were detected among F4, F5, F3 and F2; but the F4 treatment had 8.01–12.53% more accumulated dry matter than F1. At 90 days after sowing, while no significant differences were observed between F4, F5 and F3, there was 34.58–40.36% and 58.81–64.13% greater dry matter accumulation under F4 than F2 and F1, respectively. At 111 days after sowing, F4 and F5 had a similar amount of accumulated dry matter, but that of F4 was 10.55–13.53%, 35.74–39.14% and 54.64–62.73% higher than those of F3, F2 and F1, respectively. Finally, at 144 days after sowing, F4 and F5 remained indistinguishable, but for F4 there was significantly greater dry matter accumulated than for F1, F2 and F3.

The rates at which dry matter accumulated under the different fertilization treatments varied over time since sowing (Table 5). With a greater frequency of fertilization, the vm also increased. The vm of the F4 treatment was 2.94–3.03%, 51.11–59.09% and 88.89–100.00% higher than those of F3, F2 and F1, respectively. The td of the F4 treatment was 0.7–1.97, 0.35–1.91 and 2.08–2.10 days longer than those of F3, F2 and F1, respectively. In summary, applying nitrogen in small amounts but at multiple times was able to increase the dry matter growth rate, and extend its duration, which promoted grain formation and augmented the yield of maize.

**Table 5:** Parameters of dry matter characteristics for maize in both years of the experiment

Year	Treatment	Parameter				
		*t*_1_	*t*_2_	vm	tm	td
2020	F0	57.33	90.07	0.33	76.70	32.74
	F1	53.68	88.59	0.35	73.64	34.91
	F2	55.82	90.90	0.44	73.86	35.08
	F3	57.49	92.51	0.68	74.50	35.02
	F4	59.62	95.61	0.70	78.12	36.99
	F5	60.99	95.62	0.72	77.31	35.63
2021	F0	56.87	90.97	0.34	75.42	33.10
	F1	53.67	87.84	0.36	72.76	34.17
	F2	54.28	89.20	0.45	70.74	35.92
	F3	57.68	92.25	0.66	72.45	35.57
	F4	59.01	95.28	0.68	76.15	36.27
	F5	59.21	95.14	0.68	76.18	36.93

### Material accumulation, Na and harvest index (HI)

Both the matter accumulation and Na in maize were affected significantly by the nitrogen application frequency (Table 6). In both years, the pre‐anthesis accumulations in stems and leaves lacked any significant difference among the F2, F3, F4 and F5 treatments, but they significantly surpassed those of the F1 and F0 treatments. For the post‐anthesis accumulations, the F4 and F5 treatments were similar in their Na in stems and leaves, as well as HI and Na; however, these values were significantly higher than those of F3, F2, F1 and F0. The nitrogen transfer rate from stems and leaves to grains diminished with more nitrogen applications. The Na in the F4 treatment was 21.46–32.14%, 54.17–54.60% and 86.75–89.20% greater than in F3, F2 and F1, respectively. The HI in the F4 treatment was 5.66%, 9.80% and 12.00% higher than in F3, F2 and F1, respectively.

**Table 6:** Pre‐anthesis and post‐anthesis material accumulation, HI and amount of nitrogen accumulation in maize

Year	Treatment	Pre‐silking DM (t ha^−1^)	Na at silking stage (kg ha^−1^)		Post‐silking DM (t ha^−1^)	Na at maturity stage (kg ha^−1^)		Nitrogen transport rate (%)		Na (kg ha^−1^)	HI
			Stem	Leaf		Stem	Leaf	Stem	Leaf		
2020	F0	9.50c	34.07c	76.12c	9.91e	4.10e	6.89e	87.96a	90.95a	101.68e	0.48e
	F1	10.45b	45.40b	87.95b	11.37d	7.29d	9.28d	86.84a	90.53a	146.91d	0.50d
	F2	12.58a	53.68a	98.01a	13.46c	12.28c	13.19c	78.34b	86.54b	180.30c	0.51c
	F3	12.61a	54.03a	97.77a	16.49b	18.97b	23.90b	64.89c	73.37c	228.85b	0.53b
	F4	12.59a	52.79a	96.63a	20.15a	20.82a	30.55a	60.57d	67.71d	277.96a	0.56a
	F5	12.81a	52.19a	97.44a	20.19a	21.22a	30.27a	59.35d	66.89d	279.38a	0.57a
2021	F0	8.79c	45.06c	52.92c	10.16e	3.78e	5.12e	91.61a	90.33a	92.02e	0.49e
	F1	11.2b	57.29b	61.93b	11.36d	6.31d	6.67d	90.63a	91.33a	141.75d	0.50d
	F2	13.14a	69.12a	70.00a	13.81c	9.68c	9.73c	85.99b	86.11b	171.23c	0.51c
	F3	13.12a	67.22a	68.48a	15.35b	13.81b	13.74b	79.46c	79.94c	200.34b	0.53b
	F4	12.98a	68.82a	70.04a	18.69a	20.56a	21.60a	71.03d	69.16d	264.72a	0.56a
	F5	12.83a	67.73a	67.82a	19.21a	21.27a	20.82a	68.60d	70.78d	262.12a	0.56a

**Figure 6:**
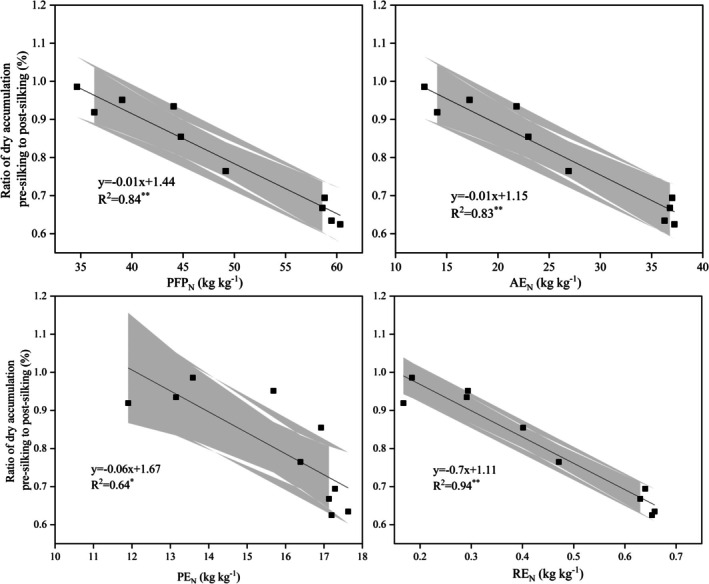
Relationships between the ratio of dry matter accumulation in the pre‐silking to post‐silking and PFPN (partial factor productivity of applied nitrogen) or AEN (agronomic nitrogen‐use efficiency) or PNUE (physiological nitrogen‐use efficiency) or NRE (nitrogen‐recovery efficiency). The gray shading represents the 95% confidence intervals for the fitted regression line.

The before‐ to after‐flowering ratio had significant negative correlation with PFPN, AEN, PEN or REN (Fig. 6). This distinction clearly pointed to a trade‐off between vegetative growth and reproductive production in maize.

### Correlation analysis

**Figure 7:**
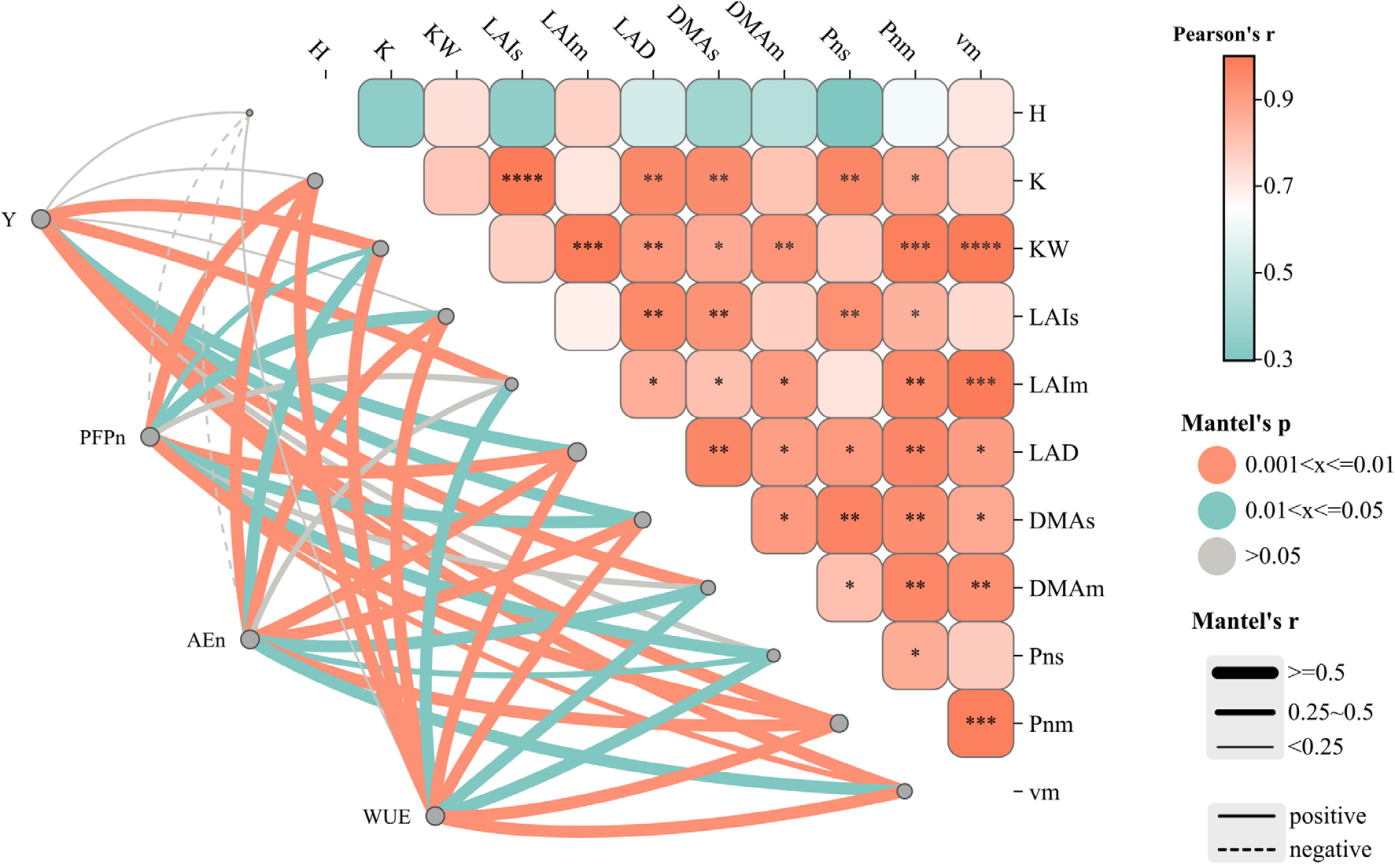
Correlation analysis between the yield (Y), PFPN, AEN, WUE of maize and its number of harvested ears (H), number of grains per ear (K), 1000‐grain weight (KW), LAI at silking and maturity stages (LAIs and LAIm, respectively), pre‐ and post‐anthesis dry matter accumulation (DMAs and DMAm, respectively), photosynthetic rate at silking and dough stages (Pns and Pnm, respectively), maximum dry matter accumulation rate (vm) and harvest index (HI).

It was found that KW had a significant positive correlation with DMAs, DMAm, LAIm, LAD, Pm, HI and vm (*P* ≤ 0.05), as did K with LAIs, LAD, DMAs, Pns and Pnm (Fig. 7). According to the Mantel analysis, KW, LAIm, LAD, Pnm, DMAm, vm and HI were positive driving factors of maize yield. Furthermore, PFPN was positively influenced by K, KW, LAIs, LAD, DMAs, Pns, Pnm, vm and HI; PEN was positively influenced by K, KW, LAIs, LAIm, LAD, DMAs, DMAm, Pnm, vm and HI; and REN was positively influenced by K, LAIs, LAIm, LAD, DMAs, DMAm, Pnm, vm and HI. In addition, AEN and WUE were positively driven by all the indicators except H. These results indicated that a differing frequency of nitrogen application mainly affected the post‐anthesis photosynthetic rate and material accumulation rate of maize by influencing its LAI and biomass after anthesis, thereby altering the 1000‐grain weight and further influencing the yield and water‐/fertilizer‐use efficiency of maize.

### Working model of optimizing fertilization frequency

**Figure 8:**
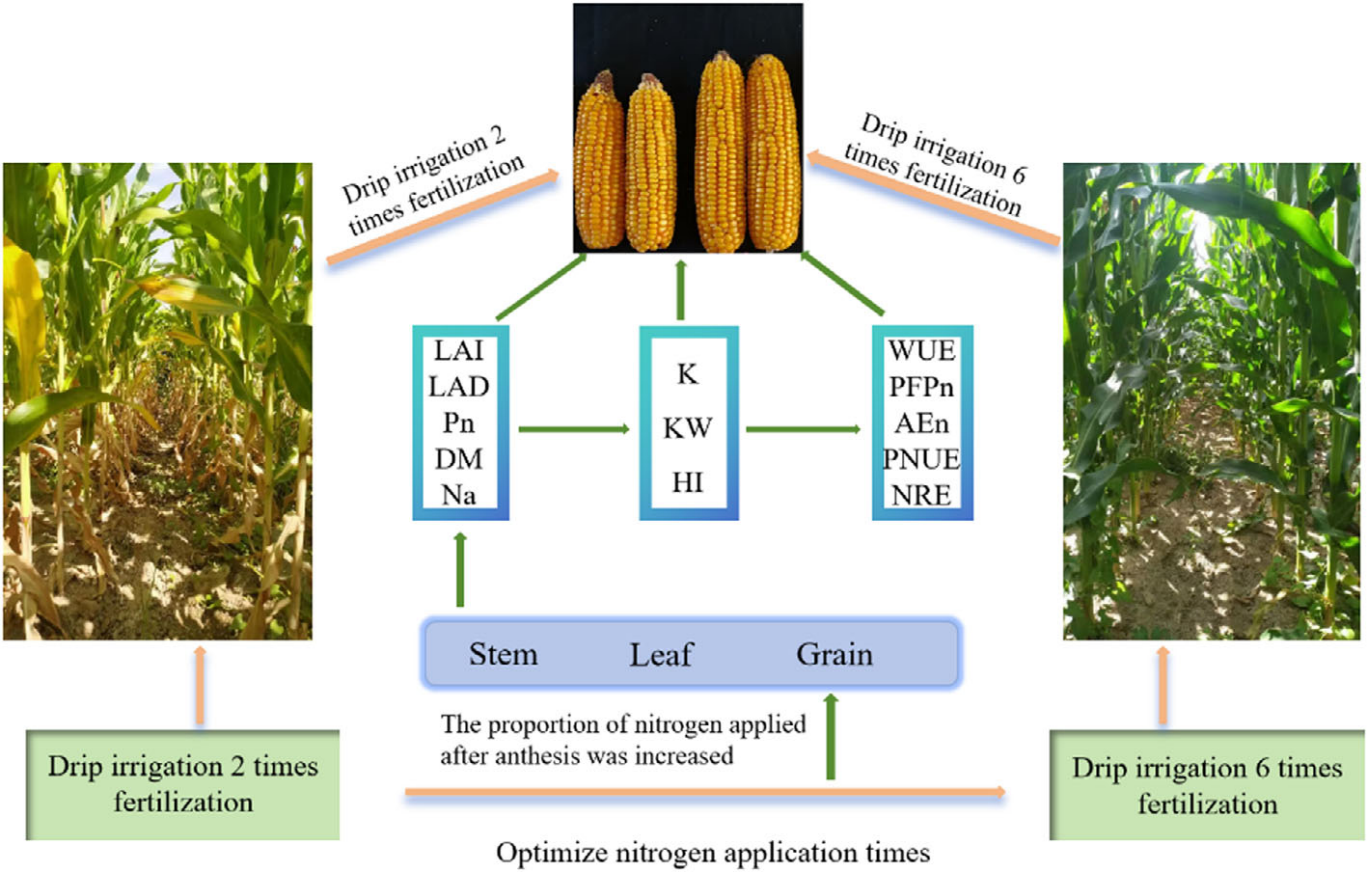
Working model of drip irrigation to simultaneously improve the biomass of maize and its water and nitrogen utilization by optimizing fertilization frequency.

Optimizing fertilization frequency and the proportion of nitrogen applied after anthesis was increased (Fig. 8). By increasing the LAI, dry matter accumulation (DMA) and Na, the number of grains per spike (K) and the grain weight (KW) can be increased, and the yield as well as the water‐/nitrogen‐use efficiency can be improved.

## DISCUSSION

### Effects of nitrogen application frequency on yield and nitrogen‐use efficiency of maize

Under the drip irrigation and fertilization integrated technique, applying nitrogen fertilizer in small but multiple doses is capable of meeting the nutrient needs of crops, increasing maize yields and enhancing fertilizer utilization rates.^23^, ^24^ The reasonable allocation of nitrogen fertilizer during maize's entire growth period is crucial for increasing both its material accumulation and yield, while also improving the nitrogen fertilizer utilization efficiency and reducing adverse environmental impacts like nitrogen leaching.^25^ In this study, using that integrated mode of technology and increasing the frequency of nitrogen top dressing synchronized crop demand with nutrient supply, which improved nitrogen availability for maize and benefited crop growth and yield formation. The greatest yield was attained using the six‐time nitrogen top dressing at 15.82–16.06 t ha^−1^, with a PFPN of 58.81–60.33 kg kg^−1^ (Table 1). Compared with either a four‐time or two‐time fertilization, the yield increased by 22.53–31.35% or 35.61–50.66%, respectively. In previous maize nitrogen fertilizer research done in the same region, with nitrogen top dressing only at the basal and jointing stages, the greatest yield was only 10.30–11.06 t ha^−1^, with PFPN of 45.00–46.40 kg kg^−1^.^26^ Therefore, the obtained yield in the present study was 45.21–53.69% higher, while PFPN was 30.02–30.69% higher. The possible reason for this disparity is that if the nitrogen application frequency is too low, the excess nitrogen cannot be absorbed and utilized by maize all at once; hence it is readily hydrolyzed into ammonium substances that flow to deep soil or get volatilized with water movement. This would lead to a nitrogen deficiency in the late growth stage, affecting yield and nitrogen fertilizer productivity.^27^ Additionally, in 2020, the light and temperature conditions were favorable for maize growth,^21^ whereas in 2021, more rain and less sunlight during the silking stage likely slowed maize growth, resulting in a lower yield than in 2020. The difference in yield among treatments in 2020 was smaller than in 2021, suggesting that staggering the nitrogen top dressing can improve nitrogen‐use efficiency in years considered unfavorable for maize growth.

Improving both irrigation and fertilization management to boost crop productivity is vital for addressing current and future water shortages in agriculture. Drip‐irrigation fertilization can synchronize the water and nitrogen supply with crop demand, thus offering the potential to enhance both water‐use efficiency and the nitrogen fertilizer utilization rate.^28^ In this study, a drip irrigation system was used to irrigate the maize crop with fertilization combining water and fertilizer. Appropriately increasing the fertilization frequency and reducing the irrigation volume boosted the maize yield. Compared with F1, the F4 treatment in this study saved 66.7% of irrigation water, having the best overall WUE (2.10–2.14 kg m^−3^) and yield (15.88–16.26 t ha^−1^). Excessive irrigation may not only fail to increase crop yield but also cause resource wastage, soil salinization and soil fertility decline.^29^ By contrast, drip irrigation precisely controls the water volume, reducing evaporation as well as deep percolation.^30^ Compared with traditional flood irrigation approaches, an integrated drip‐irrigation fertilization technique has significant water‐saving and yield‐increasing effects.^31^ In similar‐area irrigation research, a maize yield of 13.54 t ha^−1^ and a WUE of 1.84 kg m^−3^ were reached with a two‐time fertilization under drip irrigation.^32^ By way of comparison, the six‐time fertilization under drip irrigation tested in this study had an 18.68% higher yield and 15.21% higher WUE. The main reason being that a drip‐irrigation fertilization integration approach with a staggered fertilizer application can transport water and nutrients to a crop's roots as needed, via pressure pipes for effective absorption, to improve its yield and water‐/fertilizer‐use efficiency.

### Effect of nitrogen application frequency on matter accumulation in maize

Maize yield is affected by dry matter accumulation. An appropriate population‐level LAI is the basis of a crop population's material production.^33^ High‐yield maize's LAI first increases, stabilizes and then decreases. The split‐application of nitrogen can prolong LAI's high‐value duration and delays leaf senescence.^34^ This study found that increasing the post‐silking nitrogen application proportion helps to maintain a relatively high leaf area, delaying LAI's decline over time (Fig. 2). Post‐silking dry matter accumulation is critical for achieving a robust maize grain yield because most of the grain dry weight comes from post‐silking's photosynthetic products.^35^, ^36^ Optimizing nitrogen fertilizer management can slow late‐stage crop leaf senescence and increase late‐stage dry matter accumulation,^37^ consistent with this study's results. Under water/fertilizer integration, increasing nitrogen‐use efficiency enhances the post‐silking retention of leaf greenness, improves the LAI and photosynthetic potential from silking to physiological maturity, and reduces the leaf senescence rate of maize (Table 4). Collectively, that promoted its population's dry matter accumulation. This shows that increasing the nitrogen top‐dressing frequency can improve maize's post‐silking photosynthetic performance, to derive more photosynthetic products, and thus increase yield. This study also found that increasing the nitrogen top‐dressing frequency effectively prolonged the rapid dry matter accumulation termination period, with the duration extended by 2.19 days and the maximum accumulation rate increased (Table 5). So, under water/fertilizer integration, suitable nitrogen fertilizer postponement can delay post‐silking leaf senescence, prolong the duration of rapid dry matter accumulation and increase the latter's rate,^38^ thereby increasing dry matter accumulation, which improves maize grain yield and the nitrogen‐use rate.

In this study, under the integrated water–fertilizer mode of management, with multiple fertilization and an increased post‐anthesis fertilization proportion, the ratio of pre‐ to post‐anthesis was negatively correlated with PFPN, AEN, PEN and REN (Fig. 6). This shows the coordination between maize's vegetative and reproductive growth. Appropriately postponing the application of nitrogen can satisfy the late‐stage nitrogen demand of maize, enhance nitrogen‐use efficiency and boost grain‐filling rate and period to affect the final yield.^39^ Post‐anthesis matter accumulation and LAI were positively correlated with the maize grain weight (Fig. 7).

However, continuing to increase the frequency of fertilization (F5) has no significant impact on maize yield, which suggests there are some limitations to the fixed‐quantity fertilization, with the proportion of nitrogen fertilizer allocation still unclear. Looking ahead, it is necessary to conduct research on the allocation of nitrogen fertilizer in different growth stages of maize to further clarify the impact of nitrogen fertilizer allocation on yield and nitrogen fertilizer utilization rate, so as to provide timely technical guidance for strengthening the nitrogen application strategy of maize for supplementary irrigation areas in northeast China.

## CONCLUSION

Applying nitrogen six times achieved the best yield of maize (ranging from 15.82 to 16.06 t ha^−1^) and the optimal nitrogen utilization rate (with an AEN of 36.78–37.22 kg kg^−1^, PFPN of 37.0–37.22 kg kg^−1^, PEN at 7.44–7.87 kg kg^−1^ and REN at 48.12–50.35 kg kg^−1^). The WUE reached 2.10–2.14 kg m^−3^. Compared with the four‐time and two‐time fertilization modes typically used by farmers, the yield of maize increased by 22.53% to 31.35% and 35.61% to 50.66%, respectively. More frequent nitrogen applications can prolong the functional period of maize leaves, slow down leaf senescence, increase their photosynthetic utilization rate, enhance post‐anthesis matter accumulation, increase the weight of maize kernels, augment the amount of nitrogen accumulated and improve both water‐ and nitrogen‐use efficiency of maize, thereby raising the maize yield.

## AUTHOR CONTRIBUTIONS

GZ, KW, RX and SL conceived and designed the experiment; DS, LZ, LF, ZW, TZ, JY, XX, HJ and NM performed the experiments; DS, GZ, BM and JX analyzed the data; DS wrote the paper; GZ and SL revised the final draft manuscript. All authors have read and agreed to the published version of the paper.

## CONFLICT OF INTEREST

The authors declare that there are no conflicts of interest.

## Data Availability

The data presented in this study are available on request from the corresponding author.
